# Association of Gut Microbiota and Biochemical Features in a Chinese Population With Renal Uric Acid Stone

**DOI:** 10.3389/fphar.2022.888883

**Published:** 2022-05-19

**Authors:** Cheng Cao, Bo Fan, Jin Zhu, Na Zhu, Jing-Yuan Cao, Dong-Rong Yang

**Affiliations:** ^1^ Department of Urology, The Second Affiliated Hospital of Soochow University, Suzhou, China; ^2^ Department of Urology, Changshu Hospital Affiliated to Soochow University, Changshu, China; ^3^ Department of Rheumatology, Changshu Hospital Affiliated to Soochow University, Changshu, China; ^4^ Department of Nephrology, Taizhou People’s Hospital, Taizhou, China

**Keywords:** gut microbiota, uric acid stone, nephrolithiasis, 16srRNA, biomarker, computational intelligence

## Abstract

Previous studies suggest that patients with nephrolithiasis exhibit dysbiosis in their gut microbiota, but those studies were conducted in calcium oxalate stone patients. We aimed to explore the association of gut microbiota and biochemical features of renal uric acid stone (UAS) patients in a Chinese population and identify the related bacteria that may affect the pathopoiesis of UAS. A case-control study of 117 patients with UAS, 123 patients with gout, and 135 healthy controls were included from January 2014 to October 2020. For each subject, data on demographics, biochemical parameters of blood and urine were analyzed. Fifteen patients with gout, 16 patients with UAS, 17 UAS patients with gout, and 17 healthy subjects were enrolled and provided fecal samples. The characteristics of gut microbiota were explored by using 16S ribosomal RNA (rRNA) gene sequencing and analyzed by using a combination of software mother and R. Hyperuricemia was the main risk factor for the development of gout and UAS. Obesity, dyslipidemia, and aciduria were unique risk factors for UAS patients. The richness, diversity, and relative abundance of dominant bacteria at the phylum and genus levels of gut microbiota in UAS patients were significantly distinct from other subjects. Abundance of *Bacteroides* and *Fusobacterium* was significantly positively correlated with the serum uric acid (UA) level of UAS patients. Fusobacteria was involved in the metabolism and degradation of certain short-chain fatty acids, amino acids, and sugars in pathopoiesis of UAS, and inhibited their synthesis pathways. Fusobacteria may be related to the pathogenesis of UAS, and this finding contributes to the personalized treatment of UAS from the perspective of maintaining micro-ecological equilibrium in gut.

## Introduction

Nephrolithiasis is a global disease across regions and ethnicities, which is considered an important public health problem. It has a severe impact on human health and causes a huge social and economic burden (Sakhaee, 2008). To make matters worse, the recurrence rate of nephrolithiasis remains high, with a recurrence rate of 50% within 10 years. Renal uric acid stone (UAS) is indicated in the presence of a radiolucent stone (Ganesan et al., 2018), accounting for 10–15% of all urinary calculi, and its prevalence rate varies globally (Abou-Elela, 2017; Trinchieri and Montanari, 2017). The recurrence rate of patients with UAS is close to 100% (Wang et al., 2017). Although no large-scale epidemiological studies have been conducted, the prevalence of UAS in China has increased significantly in the past 30 years. In the economically developed areas of southern China, the prevalence of UAS is as high as 12–18% (Ma et al., 2018).

The microbiota of the human intestinal tract is a complex community composed of more than 100 trillion microbial cells, including more than 1,000 different kinds of species (De Sordi et al., 2017). High-throughput sequencing has facilitated great advances in our understanding of gut microbiome. Gut microbiota is increasingly linked to the development of various metabolic diseases such as obesity, diabetes, dyslipidemia, kidney disease, and nephrolithiasis (Langille et al., 2013; Stern et al., 2016; Stanford et al., 2020). The discovery of Oxalobacter formigenes, which is a kind of oxalate degrading bacteria, makes it possible that the gut microbiota affects absorption and secretion of solutes relevant to kidney stone formation (Siva et al., 2009). To date, more studies on the relationship between gut microbiota and nephrolithiasis are reported (Tang et al., 2018). They found that the cause of calcium oxalate stone was related to a group of bacteria involved in oxalate degradation and transportation, rather than a single kind of bacteria (Lee and Stern, 2019). The relationship of gut microbiota and calcium oxalate stone has been investigated in a limited amount (Stern et al., 2016; Lee and Stern, 2019), especially with no study of gut microbiota and UAS, to the best of our knowledge.

Although hyperuricemia is the common physiological and pathological bases of UAS and gout, only part of the population will develop UAS or gout, and the mechanism is still being studied. Patients with a history of gout have greater risk of forming UAS, and patients with obesity, diabetes, dyslipidemia, or other metabolic syndrome (Joosten et al., 2020). However, many patients with UAS are not accompanied by gout. The loss of bicarbonate leads to the formation of acidic urine, which is the most direct risk factor for UAS. Gastrointestinal diseases such as inflammatory bowel disease have been shown to cause bicarbonate loss clearly, and we have the reason to believe that gut microbiota and its metabolites regulate urine pH. We attempted to explore the differences in biochemical and gut microbiota features between UAS and gout patients, and find out bacteria genera, which involved in the pathogenesis of UAS. Through the analysis and detection of target bacteria by computational intelligence, it facilitates a new thinking for the personalized diagnosis and treatment of UAS patients.

## Materials and Methods

### Human Study Designs, Subjects, and Sampling

This study was approved by the Ethics Review Committee of Changshu Hospital affiliated to Soochow University. All the participants were local residents of unrelated southern Han Chinese. A case-control study of 123 gout patients (Gout group), 87 UAS patients without gout (UAS group), 30 UAS patients complicated with gout (Gout + UAS group), and 135 stone-free healthy people (Control Group) who received physical examination in Changshu Hospital affiliated to Soochow University from January 2014 to October 2020 were conducted. All the stone patients were diagnosed by ultrasound of the urinary system or abdominal-computed tomography, and received ureteroscopy or percutaneous nephrolithotomy. The stones obtained after operation were identified as pure or mixed uric acid calculus (uric acid content is greater than 50%) by Fourier-transformed infrared spectrophotometry LIIR-20 (Lanmode scientific instrument Co., Ltd., Tianjin, China). Patients who had a history of statins, chronic liver insufficiency, malignant tumors, and thyroid or parathyroid diseases were excluded from the study. Controls with a history or any evidence of nephrolithiasis, self-reported history of dyslipidemia or use of statins were excluded from the study.

General body measurements (height, weight, and blood pressure), past medical history, and test of blood and urine were carried out in all subjects. About 5 ml fasting venous blood sample and mid-stream urine of the first urine in the morning were drawn from each subject. Under the matching of age, sex, and BMI, 15 patients in the Gout group, 16 patients in the UAS group, 17 patients in the Gout + UAS group, and 17 patients in the controls were selected (Supplementary Table S1). All subjects were long-term residents with similar dietary habits, and underwent food frequency questionnaire before enrollment. After 3 days of eating uniform diet provided by the hospital (Supplementary Table S2), fresh fecal samples from the aforementioned people were collected, froze immediately, and stored under −80°C until analysis. Subjects who support fecal samples were excluded if they used antibiotics within 3 months, had a history of chronic diarrhea or constipation, chronic enteritis, irritable bowel syndrome, gastrointestinal tumor, or intestinal surgery.

### 16s DNA Extraction, PCR Amplification, and Target Gene Sequencing

The DNA was extracted from 200 mg samples using the QIAamp DNA fecal mini kit (QIAGEN, Hilden, Germany) following the manufacturer’s instructions. DNA was checked by running the samples on 1.2% agarose gels.

The V3–V4 hyper-variable regions of the bacteria 16SrRNA gene were amplified with primers 357F (5′-ACTCCTACGGRAGGCAGCAG-3′) and 806R (5′-GGACTACHVGGGTWTCTAAT-3′) by the polymerase chain reaction (PCR) system. Prior to library pooling, the barcoded PCR products were purified using a DNA gel extraction kit (Axygen, United States) and quantified using the FTC-3000 TM real-time PCR (Funglyn Shanghai). The PCR products from different samples were indexed and mixed at equal ratios for sequencing on the illumina platform at TinyGen Bio-Tech (Shanghai) Co., Ltd.

### Statistical and Bioinformatics Analysis

The general characteristics and biochemical parameters of the subjects were analyzed by SPSS22.0 and 2-sided *p* < 0.05 was defined as statistically significant. Continuous variables were summarized with mean and standard deviation if they satisfied the homogeneity of normality and variance. Otherwise, they were reported with median and inter quartile range (IQR). The quantitative variables were tested by the analysis of variance (ANOVA), Kruskal–Wallis test, and Mann–Whitney test. The chi-square test was used for categorical variables. Multiple logistic regression analysis was used to analyze the risk factors of disease in each group, and OR values were adjusted according to age and sex.

16S sequences were analyzed using a combination of software mother and R. The demultiplexed reads were clustered at 97% sequence identity into operational taxonomic units (OTUs) and the singleton OTUs were deleted using the UPARSE pipeline. OTU taxonomies were determined based on the NCBI. Based on taxonomic information, the community structure was statistically analyzed from the classification level of phylum, class, order, family, genus, and species. For the alpha-diversity analysis, Shannon, Simpson, Chao, ACE index, and rarefaction curves were calculated using mothur and plotted by R. The Kruskal–Wallis test was used to detect the significant changes of Shannon, Simpson, Chao, and ACE index between each group. For the beta-diversity metrics, the weighted UniFrac distance matrix were calculated using mothur and visualized with principal coordinate analysis (PCoA) by ape package in R. Linear discriminant analysis effect size (LEfSe) analysis was performed. Linear discriminant analysis was performed on samples with different grouping conditions according to the taxonomic composition. LDA was used to screen the communities or species which had significant influence on the differences in sample division with a cutoff of 2.0. The Spearman correlation analysis was used to evaluate association of dominant bacteria genera and biochemical parameters. The metabolic pathways of Kyoto Encyclopedia of Genes and Genomes (KEGG) were predicted by PICRUST software package.

## Results

### General Characteristics of the Study Population

The proportion of hypertensive patients and body mass index (BMI) in observation groups were significantly higher than the controls. Age (*p* = 0.571), gender (*p* = 0.310), and prevalence of diabetes mellitus (*p* = 0.413) were not significantly different among each group. However, BMI of the two stone groups, especially the Gout + UAS group was significantly higher than the other groups (*p* < 0.001). More hypertension individuals were found among the three observation groups and the Gout + UAS group is still with the highest prevalence rate (*p* < 0.001) (Table1).

**TABLE 1:** Comparison of general characteristics between each group.

Variables	Gout	UAS	Gout + UAS	Control	*p* value
Age, years	61.46 ± 15.58	60.32 ± 14.74	57.77 ± 14.39	59.25 ± 16.56	0.571^#^
Gender (%)					0.310^*^
Male	105 (85.37)	67 (77.01)	26 (86.67)	106 (78.52)	
Female	18 (14.63)	20 (22.99)	4 (13.33)	29 (21.48)	
BMI	23.88 ± 2.46^b^	24.92 ± 3.53^a^ ^,^ ^c^	26.80 ± 2.19^d^	23.75 ± 1.51	**<0.001** ^**#**^
Hypertension (%)					**<0.001** ^*^
Yes	54 (43.90)^d^	36 (41.38)^c^	17 (56.67)^c^	31 (22.96)	
No	69 (56.10)^d^	51 (58.62)^c^	13 (43.33)^c^	104 (77.04)	
Diabetes (%)					0.413^*^
Yes	7 (5.69)	6 (6.90)	4 (13.33)	7 (5.19)	
No	116 (94.31)	81 (93.10)	26 (86.67)	128 (94.81)	

#Analysis of variance (ANOVA)

*2-sided Chi-square test.

a *p* < 0.05 (compared with the Gout + UAS, group).

b *p* < 0.001 (compared with the Gout + UAS, group).

c *p* < 0.05 (compared with the control group).

d *p* < 0.001 (compared with the control group).

UAS, uric acid stone; BMI, body mass index.

Bold values indicate significant difference.

### Biochemical Features and Risk Factors Analysis Among the Groups

Hyperuricemia was the main risk factor for the development of gout as well as UAS. Obesity, hypertriglyceridemia, and aciduria were unique risk factors for UAS patients. Main biochemical features are summarized in Table 2 or Supplementary Figure S1. Compared to the controls, the triglycerides (TG) level in the three observation groups was significantly higher, and the level of TG in the Gout + UAS group was the highest (*p* < 0.05). The level of high-density lipoprotein cholesterol (HDL-C) in Gout as well as Gout + UAS group was significantly lower than that in the control group. The concentration of potassium in the Gout and UAS groups was significantly lower than that in the Gout + UAS group (*p* < 0.05), and the serum calcium concentration in the Gout group was also significantly lower than the Gout + UAS group and the controls (*p* < 0.05). All the three observation groups have significantly higher serum uric acid (UA) value than the controls (*p* < 0.001), and among them the Gout + UAS group’s UA value was the highest (*p* < 0.001). All the patients in three observation groups demonstrated obvious aciduria, especially in the Gout + UAS group (Table2, Supplementary Figure S1).

**TABLE 2:** Biochemical parameters in different groups studied.

Variables	Gout	UAS	Gout + UAS	Control
Lipid levels, mmol/L				
TG	1.74 ± 1.08^a^ ^,^ ^c^	1.81 ± 0.98^a^ ^,^ ^d^	2.66 ± 1.97^c^	1.37 ± 0.61
TC	4.66 ± 0.98	4.83 ± 0.87	4.77 ± 1.23	4.83 ± 0.98
HDL-C	1.18 ± 0.30^c^	1.23 ± 0.35^a^	1.06 ± 0.25^d^	1.31 ± 0.34
LDL-C	2.60 ± 0.69	2.77 ± 0.63	2.72 ± 0.81	2.77 ± 0.71
Electrolyte levels, mmol/L				
K	4.04 ± 0.37^a^ ^,^ ^c^	4.08 ± 0.41^a^	4.33 ± 0.62	4.15 ± 0.42
Na	140.31 ± 3.37	140.95 ± 2.54	140.94 ± 2.74	140.79 ± 2.18
Cl	103.95 ± 3.39	104.54 ± 3.23	105.31 ± 3.46	104.24 ± 2.98
Ca	2.29 ± 0.15^a^ ^,^ ^c^	2.34 ± 0.16	2.38 ± 0.16	2.35 ± 0.13
P	1.07 ± 0.24	1.05 ± 0.22	1.12 ± 0.36	1.03 ± 0.18
Mg	0.86 ± 0.10	0.87 ± 0.10	0.89 ± 0.20	0.88 ± 0.08
UA (μmol/L)	465.54 ± 126.22^b^ ^,^ ^d^	392.71 ± 93.12^b^ ^,^ ^d^	559.13 ± 76.57^d^	336.09 ± 76.75
Urinary pH	5.72 ± 0.55^d^	5.64 ± 0.55^d^	5.57 ± 0.63^c^	5.99 ± 0.67

a *p* < 0.05 (compared with the Gout + UAS, group).

b *p* < 0.001 (compared with the Gout + UAS, group).

c *p* < 0.05 (compared with the control group).

d *p* < 0.001 (compared with the control group).

UAS, uric acid stone; TG, triglycerides; TC, total cholesterol; HDL-C, high-density lipoprotein cholesterol; LDL-C, low-density lipoprotein cholesterol; and UA, serum uric acid.

Comparisons made using Student’s t test.

Univariate logistic analysis suggested that hypertension, low HDL-cholesterolemiamia, hyperuricemia, and aciduria were independent risk factors for gout. Obesity, hypertension, hypertriglyceridemia, low HDL-cholesterolemiamia, hyperuricemia, and aciduria were common independent risk factors for all UAS groups (Supplementary Table S3). Adjusted for age and sex, multivariate logistic analysis indicated that only hyperuricemia was the common risk factor for the development of gout as well as UAS. For patients with UAS alone, obesity, hypertriglyceridemia, and aciduria were the other three risk factors. While obesity was another risk factor for patients with UAS complicated with gout besides hyperuricemia (Table 3).

**TABLE 3:** Multivariate logistic analysis of risk factors in each group studied.

Variables	Gout		UAS		Gout + UAS	
	*p* value^a^	Adjusted OR (95% CI)	*p* value^a^	Adjusted OR (95% CI)	*p* value^a^	Adjusted OR (95% CI)
BMI	0.612^b^	1.03 (0.91–1.17)	**0.019**	**1.16(1.03–1.32)**	**0.015**	**12.57(1.62–97.47)**
Hypertension	0.088	1.75 (0.92–3.34)	0.066	1.93 (0.96–3.87)	0.415	0.12 (0.01–19.00)
Hypertriglyceridemia	0.154^b^	1.76 (0.81–3.82)	**0.002**	**4.19(1.67–10.40)**	0.208	13.90 (0.23–839.09)
Low HDL-cholesterolemiamia	0.080	1.96 (0.92–4.17)	0.213	1.70 (0.74–3.93)	0.051	89.59 (0.98–8.19e-3)
Hyperuricemia	**<0.001**	**10.08(5.27–19.27)**	**<0.001**	**4.81(2.30–10.07)**	**0.021**	**2.87e-6(9.74-8.46e-11)**
Urinary pH	0.122	0.68 (0.42–1.11)	**0.027**	**0.55(0.32–0.93)**	0.662	0.60 (0.06–6.09)

a Adjusted for age and sex in the multivariate logistic regression model.

b Results of univariate logistic regression analysis.

UAS, uric acid stone and BMI, body mass index.

Bold values indicate significant difference.

### 16S Sequencing Depth and Analysis of the Sample Size

**FIGURE 1:**
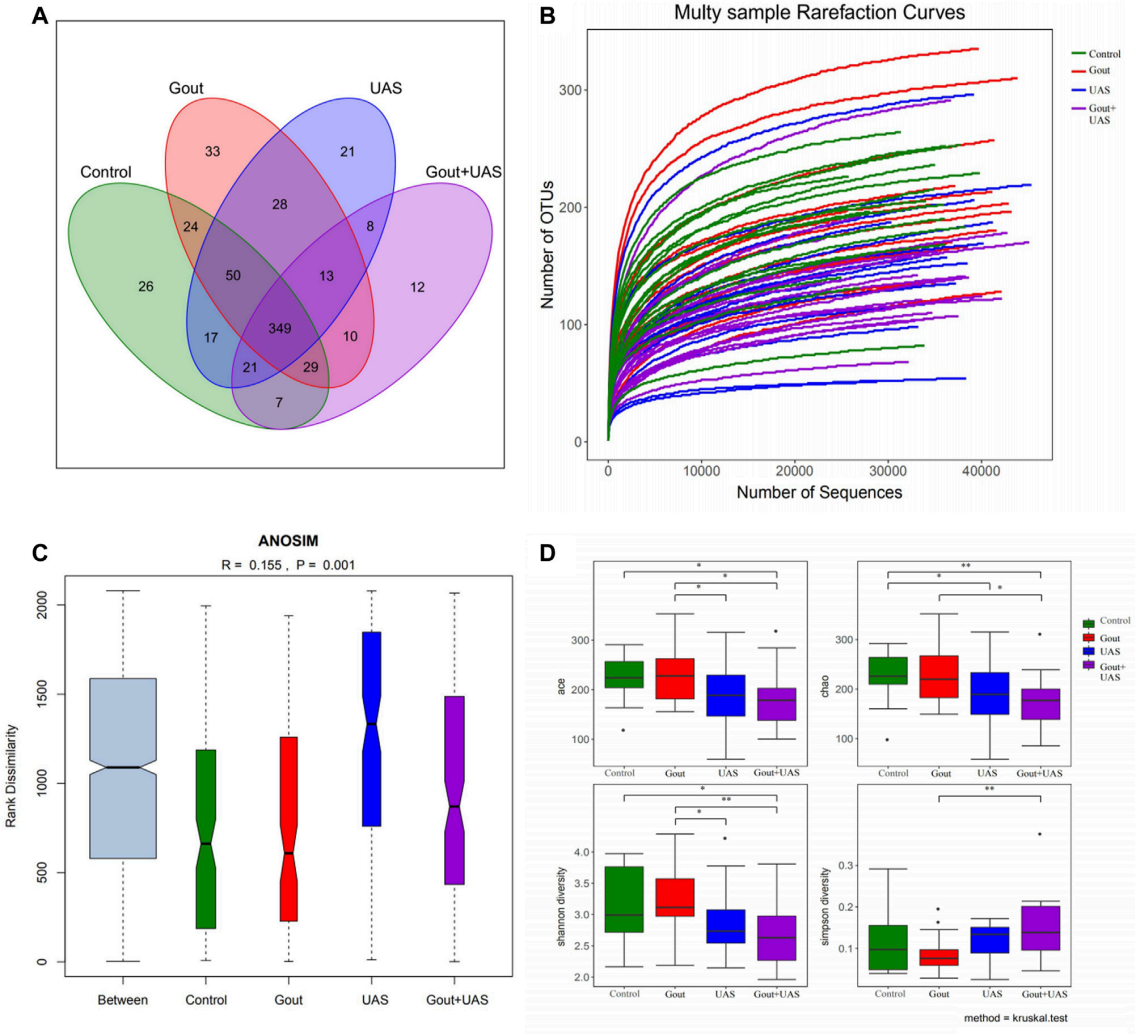
Analysis of gut microbiota among each group by using 16s rRNA. **(A)** Venn diagram for indicating the common and unique OTUs among four groups. **(B)** Multi-sample rarefaction curves for comparing the abundance of diverse species in each sample. **(C)** ANOSIM analysis for identifying the existence of differences between each group. **(D)** Comparison of alpha diversity of gut microbiota between each group. The ACE, Chao, Shannon, and Simpson indices at the operational taxonomic units (OTUs) level were compared between each group by Kruskal–Wallis test (*p < 0.05**p < 0.01).

The study was carried out with reasonable sample collection and high species rich, and the amount of data was reasonable and could reflect the majority of gut microbiota information objectively among groups. After eliminating the sequences of repetitive and fuzzy bases that affect the quality of analysis, a total of 2,329,290 high-quality sequences with an average length of 459 were received. The number of total OTUs in this study was 700, including one kingdom, 12 phylum, 21 class, 29 order, 46 family, 132 genus, and 181 species. The Venn diagram shows the common and unique OUTs among each group. Among the 648 known OUTs, the number of OUTs in the Gout + UAS group was lower than other groups (Figure 1A). With the curve reached the saturation plateau, more data contribute little to the discovery of new OUTs (Figure 1B), which means the depth of sequencing was reasonable. ANOSIM analysis calculated the relationship ranking among samples through variables, and performed the substitution test to determine whether the difference between groups was significantly different from the difference within groups. The results showed that the difference of the gut microbiota structure among the four groups was statistically significant (R = 0.155, *p* = 0.001) (Figure 1C).

### Richness and Diversity of Gut Microbiota Among Each Group

The richness and diversity of gut microbiota in UAS patients, especially in stone patients complicated with gout, were significantly lower than gout patients and controls. The richness and bacterial diversity of gut microbiota in the Gout + UAS group were significantly lower than that in the Gout and Control groups (Figure 1D) from the ACE, Chao, Shannon, and Simpson indices by the Kruskal–Wallis test. PCoA analysis indicated that there are significant differences in microbiota communities in specific evolutionary lineages among the four groups. (Supplementary Figure S2).

### Analysis Based on Species Information of Microbiota Between Each Group

**FIGURE 2:**
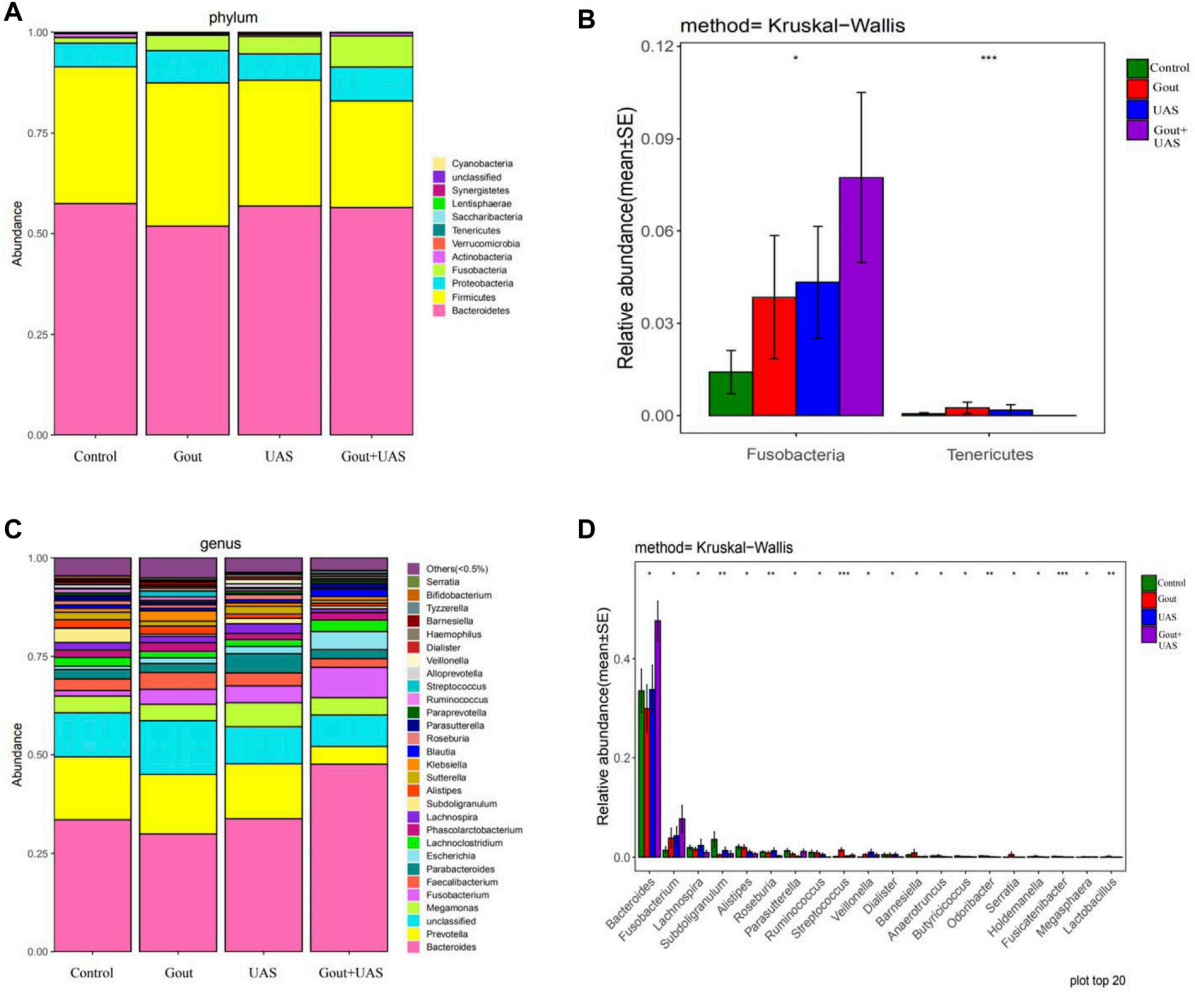
**(A,C)** Composition of gut microbiota between each group at the phylum and genus level. **(B,D)** Histograms of differences in the abundance of species analyzed by Kruskal–Wallis test among four groups at the levels of phylum and genus (*p < 0.05, **p < 0.01, and ***p < 0.001).

**FIGURE 3:**
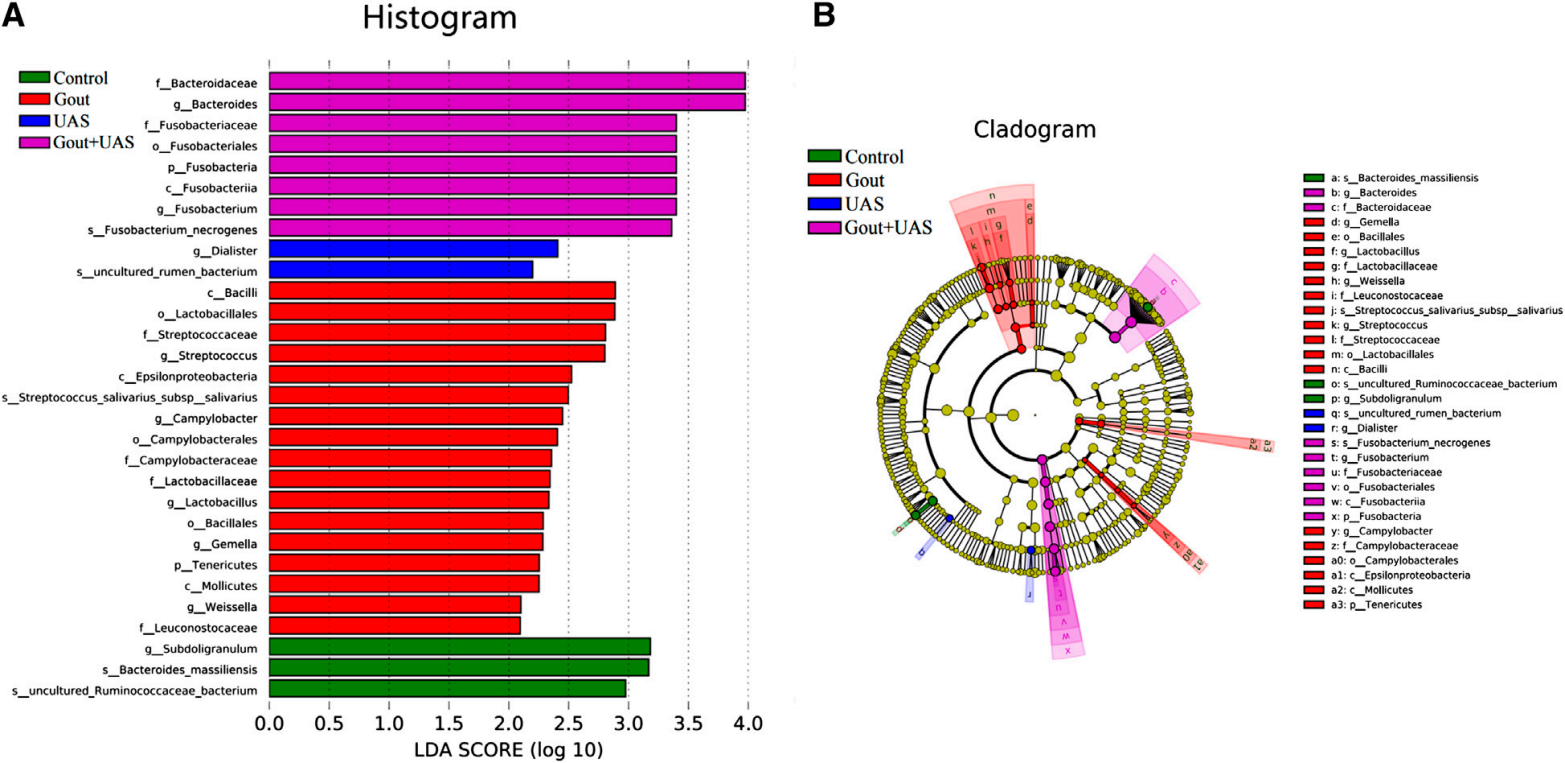
Histogram **(A)** and cladogram **(B)** of Linear discriminant analysis effect size (LEfSe) analysis based on OTUs characterizes microbiota among the controls, Gout patients, UAS patients and Gout + UAS patients.

The species diversity and relative abundance of dominant bacteria at the phylum and genus levels among the four groups were different, and certain bacteria were dominant in UAS patients. At the level of phylum, Bacteroidetes, Firmicutes, Proteobacteria, Fusobacteria, Actinobacteria were the most five abundant bacterial in each group. In the Gout + UAS group, the relative abundance of Fusobacteria was significantly higher than other groups (*p* = 0.02), while Tenericutes was significantly lower than other groups (*p* < 0.001) (Figures 2A,B). At the level of genus, the most five abundant bacterial were Bacteroides, Prevotella, Megamonas, Fusobacterium, and Faecalibacterium. The relative abundance of *Bacteroides* and *Fusobacterium* in the Gout + UAS group was significantly higher than other groups (Figures 2C,D). The linear discriminant analysis effect size diagram indicated that there were the following specific genera in each group on the phylum and genus levels Fusobacteria in the Gout + UAS group on the phylum level, Tenericutes in the Gout Group on the phylum level, *Bacteroides* and *Fusobacterium* in the Gout + UAS group on the genus level, *Streptococcus*, *Lactobacillus*, Weissella, Gemella, and *Campylobacter* in the Gout group on the genus level, *Dialister* in the UAS group on the genus level, *Subdoligranulum* in controls on the genus level (Figure 3).

### Association of Dominant Bacteria Genera and Biochemical Parameters

*Bacteroides* and *Fusobacterium* were significantly positively correlated with the serum UA level of patients with UAS. We looked for the dominant species whose LDA value was more than two and the relative abundance was in the top 20 in each group at the genus level, and screened out the following six species: *Bacteroides*, *Fusobacterium*, *Subdoligranulum*, *Streptococcus*, *Dialister*, and *Lactobacillus*. The correlation of the aforementioned genera abundance and the significant different biochemical parameters were analyzed. *Bacteroides* was positively correlated with the UA level in the Gout + UAS group (r = 0.520, *p* = 0.033), and positively correlated with the serum potassium level in the controls (r = 0.829, *p* < 0.001). *Fusobacterium* was positively correlated with the UA level in the UAS group (r = 0.560, *p* = 0.024). *Subdoligranulum* was positively correlated with the HDL-C level in the controls (r = 0.493, *p* = 0.044). *Streptococcus* was positively correlated with urinary pH in the Gout group (r = 0.536, *p* = 0.039), and inversely correlated with the TG level in the Gout + UAS group (r = -0.517, *p* = 0.034). Dialister was positively correlated with the HDL-C level (r = 0.726, *p* = 0.001), but was inversely correlated with the UA level in the UAS group (r = -0.629, *p* = 0.009) (Table 4, Supplementary Figure S3).

**TABLE 4:** | Spearman correlation coefficient of significant different biochemical parameters and major bacterial genera in each group.

Variables	*Bacteroides*		*Fusobacterium*		*Subdoligranulum*		*Streptococcus*		*Dialister*		*Lactobacillus*	
	*R*	*P*	*R*	*P*	*R*	*P*	*R*	*P*	R	*P*	*R*	*P*
Gout												
TG (mmol/L)	−0.354	0.196	0.139	0.621	0.079	0.781	−0.096	0.732	0.057	0.839	0.171	0.542
HDL-C (mmol/L)	0.374	0.170	0.295	0.286	−0.401	0.138	0.297	0.282	−0.219	0.433	−0.050	0.861
K (mmol/L)	−0.414	0.125	−0.257	0.355	0.234	0.401	−0.361	0.187	−0.241	0.374	−0.339	0.217
Ca (mmol/L)	−0.442	0.099	−0.292	0.292	0.477	0.072	−0.034	0.904	0.359	0.189	0.069	0.806
UA (μmol/L)	−0.401	0.132	−0.129	0.648	0.379	0.164	−0.329	0.232	0.007	0.980	0.070	0.803
Urinary pH	0.489	0.064	−0.075	0.790	−0.235	0.399	**0.536**	**0.039**	−0.075	0.789	0.194	0.487
UAS												
TG (mmol/L)	−0.036	0.895	0.184	0.495	0.016	0.952	0.137	0.613	−0.310	0.243	0.053	0.846
HDL-C (mmol/L)	−0.301	0.257	−0.137	0.614	−0.048	0.859	−0.218	0.417	**0.726**	**0.001**	0.021	0.937
K (mmol/L)	−0.467	0.068	−0.070	0.797	0.228	0.395	0.491	0.053	−0.179	0.507	0.084	0.757
Ca (mmol/L)	−0.493	0.052	0.146	0.589	0.332	0.208	0.207	0.442	0.090	0.740	−0.092	0.735
UA (μmol/L)	0.231	0.390	**0.560**	**0.024**	−0.022	0.937	0.026	0.924	**−0.629**	**0.009**	−0.358	0.173
Urinary pH	−0.057	0.835	−0.212	0.430	0.240	0.371	−0.172	0.525	0.352	0.181	0.249	0.352
Gout + UAS												
TG (mmol/L)	0.373	0.141	−0.103	0.694	0.201	0.438	**−0.517**	**0.034**	0.040	0.880	−0.446	0.073
HDL-C (mmol/L)	−0.248	0.338	0.293	0.254	−0.098	0.707	0.080	0.761	−0.033	0.899	0.083	0.752
K (mmol/L)	−0.012	0.963	−0.292	0.256	−0.135	0.605	−0.064	0.808	−0.217	0.402	−0.442	0.076
Ca (mmol/L)	−0.306	0.233	0.043	0.869	0.137	0.599	0.021	0.936	0.355	0.162	−0.039	0.882
UA (μmol/L)	**0.520**	**0.033**	−0.091	0.729	−0.479	0.052	−0.059	0.823	−0.122	0.642	−0.112	0.668
Urinary pH	0.160	0.539	−0.357	0.159	0.376	0.136	0.043	0.869	0.190	0.466	0.106	0.687
Control												
TG (mmol/L)	0.020	0.940	0.444	0.074	0.007	0.978	0.303	0.237	0.048	0.856	0.056	0.831
HDL-C (mmol/L)	−0.085	0.747	−0.342	0.179	**0.493**	**0.044**	−0.036	0.892	−0.096	0.715	0.255	0.323
K (mmol/L)	**0.829**	**<0.001**	0.273	0.289	0.001	0.996	0.101	0.700	−0.086	0.743	0.074	0.777
Ca (mmol/L)	0.249	0.334	−0.063	0.811	0.090	0.732	−0.376	0.137	−0.372	0.141	0.048	0.855
UA (μmol/L)	0.140	0.593	0.351	0.168	−0.234	0.366	−0.037	0.889	−0.088	0.738	−0.214	0.409
Urinary pH	−0.037	0.889	−0.213	0.412	0.033	0.900	−0.363	0.152	0.412	0.101	0.094	0.721

TG, triglycerides; HDL-C, high-density lipoprotein cholesterol; UA, serum uric acid; and UAS, uric acid stone.

Spearman correlation coefficient values were further adjusted for age and sex.

Bold values indicate significant difference.

### Prediction of Metabolic Function of Gut Microbiota

**FIGURE 4:**
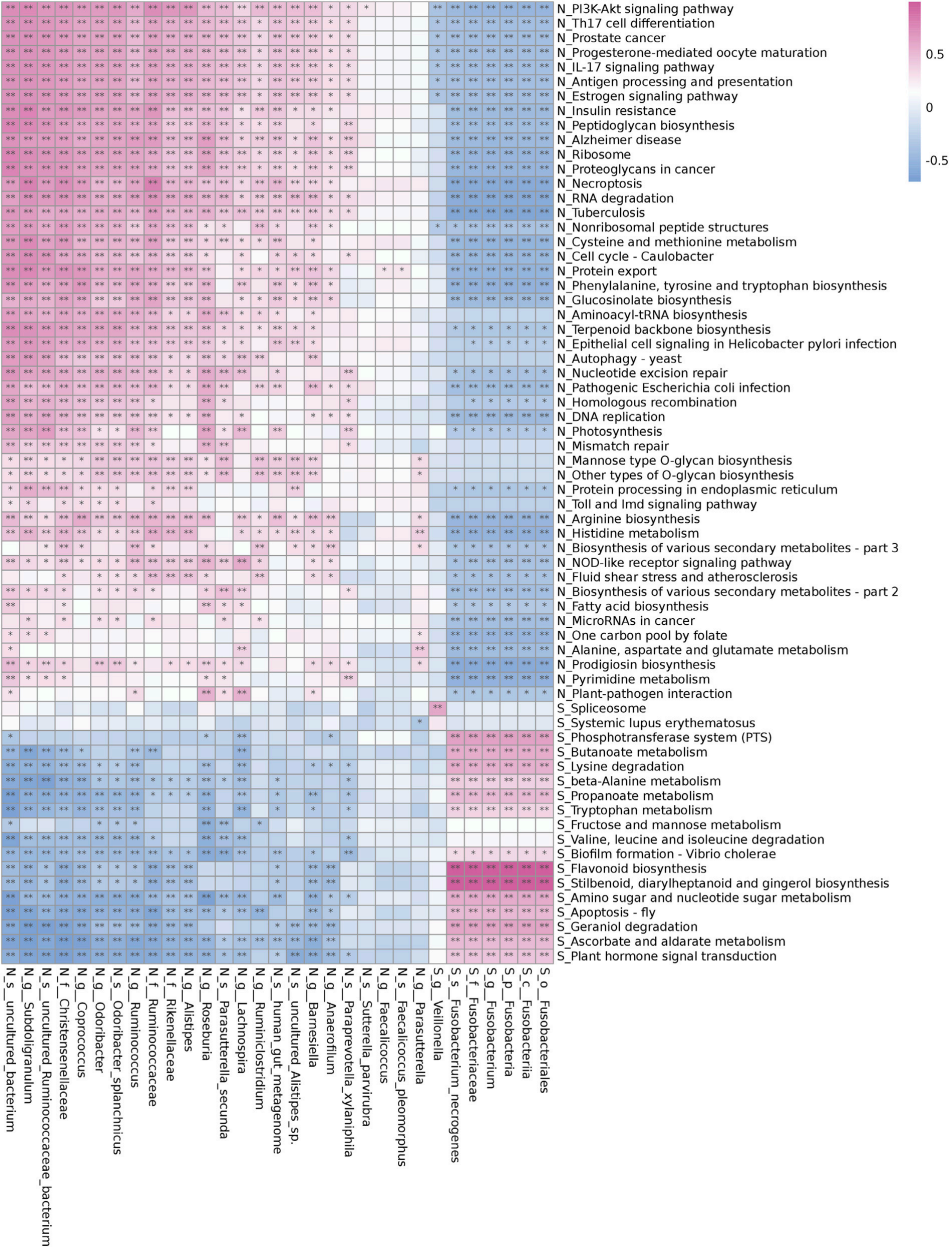
Correlation of different bacterial abundance and Kyoto Encyclopedia of Genes and Genomes (KEGG) metabolic pathways in the controls (Group N) and the renal uric acid stone patients (Group S). The red cell indicates a positive correlation and the blue cell indicates a negative correlation. Stars indicate the degree of significant correlations (*,*p* < 0.05,**,*p* < 0.01).

Fusobacteria was involved in the metabolism and degradation of certain short-chain fatty acids, amino acids, and sugars in pathogenetic of UAS, and inhibited their synthesis pathways. The PICRUSt tool was used to predict the differences in metabolic pathways between all the stone patients (Group S) and the controls (Group N) (Figure 4). Fusobacteria played a dominant role in microbiota metabolism in UAS patients and had a significant positive correlation in propanoate and butanoate metabolism, beta-alanine and tryptophan metabolism, amino sugar and nucleotide sugar metabolism, fructose and mannose metabolism, lysine, valine, leucine and isoleucine degradation and other metabolism or degradation pathway. The biosynthesis of short-chain fatty acids, the biosynthesis of phenylalanine, tyrosine, tryptophan and arginine, the biosynthesis of peptidoglycan and glucosinolate, the metabolism of cysteine, methionine, histidine, alanine, aspartate, and glutamate were inversely correlated with Fusobacteria while they were positively correlated with probiotic bacteria genera in controls.

## Discussion

In the present study, there were significant differences in biochemical features between UAS formers and gout patients. Whether for gout or UAS patients, hyperuricemia was always a risk factor. Obesity, hypertriglyceridemia, and aciduria were the risk factors of UAS, while obesity and hyperuricemia were more associated with UAS complicated with gout. The richness and diversity of gut microbiota in UAS patients were significantly lower than those in controls. *Bacteroides* and *Fusobacterium* were the dominant species that distinguish the intestinal flora of UAS patients from normal population, and they had a significant positive correlation with the level of serum UA. Fusobacteria was mainly involved in the metabolism and degradation of certain short-chain fatty acids, amino acids, and sugars in patients with UAS, and played an important role in inhibiting their synthesis pathways.

It is well known that uric acid is a weak organic acid with an ionization constant (PKA) of 5.5, the solubility of uric acid crystals decreases sharply in acidic urine with pH less than 5.5 (Tran and Maalouf, 2020). The disturbance of Na^+^/H^+^ exchange and decrease of ammonia secretion in renal proximal tubule will reduce the urinal pH value, promoting the formation of UAS and depositing of monosodium urate monohydrate, as known as the gout crystals (Friedlander et al., 2014; Rajkumar and Pluznick, 2018).

Our study indicated that the urine pH of UAS patients, especially stone formers with gout, was significantly lower than other subjects. The diseases that cause acidic urine included obesity, cardiovascular disease, insulin resistance, and chronic diarrhea (Bobulescu et al., 2019). The baseline characteristics of UAS patients in our study were partly consistent with the aforementioned clinic features. Epidemiological investigation has demonstrated that increased metabolic syndrome related factors significantly promoted the risk of UAS (Boyd et al., 2018). Several researchers have reported and confirmed that there was an inverse correlation between BMI and urine pH (Nakajima et al., 2016). Multivariate analysis was carried out in 459 24-h urine samples of 183 adult patients with nephrolithiasis and found that hyperglycemia was associated with lower pH and higher urinary saturation with respect to uric acid, suggesting that glycemic control may be considered a target for the treatment of UAS (Maciolek et al., 2018). In addition, a cross-sectional study analyzed 24-h urine parameters and abdominal CT scan results of 98 patients with nephrolithiasis and reported that the greater abdominal visceral fat content was associated with lower urinary pH and higher risk of UAS (Patel et al., 2017). Our results suggested that the incidence of diabetes in UAS patients with gout was higher than that in other groups, but there was no statistical significance, possibly due to an insufficient sample size.

Our study revealed significant differences in the levels of TG and HDL-C between the Gout + UAS group and the other three groups, which indicated a positive correlation between the severity of dyslipidemia and the risk of metabolic disorders in patients with UAS. This conclusion was roughly similar to our previous study’s results (Ding et al., 2019).

Nephrolithiasis is a known risk factor for chronic kidney disease (CKD), while UAS formation is associated with greater CKD risk compared with other stone types. Studies of patients with nephrolithiasis in Taiwan and Saudi Arabia found that the glomerular filtration rate (GFR) in UAS patients were significantly lower than those with other types of stones (Li et al., 2018; Nassir et al., 2018). While the serum potassium concentration of the Gout + UAS group was higher than other groups, suggesting decreased GFR and impaired renal tubule potassium excretion. On the flip side, the serum calcium concentration of the gout patients was the lowest, which was related to the calcium and phosphorus metabolism disorder caused by gouty nephropathy. Interestingly, there was no significant difference in the serum potassium level between gout patients and the controls. Glucocorticoid, as one of the treatment drugs for acute gout, can promote the sodium retention and potassium excretion of distal convoluted tubules and collecting tubules.

Under normal circumstances, the gut microbiota and host remains in a dynamic balance, which participates in various physiological processes of host nutrition absorption and metabolism. The gut microbiota can also affect the urine composition of the host, so the destruction of gut microbiota may lead to the occurrence of nephrolithiasis. Ticinesi et al. studied 52 patients with calcium oxalate nephrolithiasis and 48 healthy subjects, and found that the fecal microbial diversity in the stone group was significantly lower than that of the controls (Ticinesi et al., 2018). Stern et al. collected feces and urine samples of stone and non-stone patients, concluded that *Bacteroides* genus was the most abundant in stone formers and Prevotella genus was the most abundant in the control group. They also indicated that the composition of 24-h urine seemed to be related to the abundance of gut microbiota (Stern et al., 2016). A larger sample size and open-source project on the relationship between Oxalobacter formigenes and nephrolithiasis is being carried out in the United States (Liu et al., 2017).

Different from previous studies, we focused on the characteristics of gut microbiota in UAS patients for the first time. The healthy people rely on rich micro-ecosystem so as to maintain the necessary nutrition and metabolism, but with the aggravation of metabolic disorder, the intestinal microecology is destroyed. *Bacteroides* and Firmicutes accounted for more than 85% in the total amount of gut microbiota in our subjects, consistent with other experimental results at home and abroad (Ruan et al., 2020). However, there was a significant difference in the specific gravity of Fusobacteria at the phylum level. The relative abundance of Fusobacteria increased with the aggravation of metabolic disorders, and the specific gravity in Gout + UAS patients was the highest. At the genus level, *Fusobacterium* and *Bacteroides* were the dominant bacteria in patients with UAS, especially in the Gout + UAS group. Furthermore, Spearman correlation analysis results indicated that the abundance of *Bacteroides* and *Fusobacterium* was positively correlated with the serum UA level in UAS patients.

The involvement of the gut microbiota in multiple metabolic pathways in the host is widely recognized. Shotgun metagenomics sequencing technology is of course the most powerful approach to identify specific bacteria involved in metabolic pathways and identify gene function using the KEGG database. Liu et al. found a highly expressed bacterial gene in patients with recurrent calcium oxalate nephrolithiasis, which was involved in oxalate degradation and oxalate synthesis, and was related to high levels of urinary oxalate and acetic acid excretion (Liu et al., 2020). In this study, our PICRUSt analysis showed that Fusobacteria altered the microbial community functions, especially participated in the metabolism and degradation of certain short-chain fatty acids, amino acids, and sugars in UAS patients, but significantly inhibited their synthetic pathway. Future work including shotgun metagenomics analysis would help to confirm the specific gene functions.

Fusobacteria is a kind of anaerobic Gram-negative bacteria, which is widely colonized in human intestinal and oral mucosa and is related to the invasion of tumor cells (Kelly et al., 2018; Wang et al., 2020). There was a strong correlation between the abundance of Fusobacteria and the expression of pro-inflammatory markers such as COX-2, suggesting that Fusobacteria could create a pro-inflammatory micro-environment conducive to colorectal cancer by recruiting tumor-infiltrating immune cells (Kostic et al., 2013; Sears, 2018; King et al., 2020). Fusobacteria might potentially enhance the invasiveness of cancer cells when it existed in the micro-environment of oral tumor (Harrandah et al., 2020). In addition, studies have shown that the enrichment of Fusobacteria is related to acute appendicitis in children (Zhong et al., 2014; Rogers et al., 2016).

Our study indicated that the enrichment of Fusobacteria in the intestinal tract of UAS formers was higher than that of normal population for the first time. On one hand, hyperuricemia can improve the excretion level of uric acid in urine; on the other hand, it can cause dysfunction of the function of renal tubules to secrete acid and then form stones. The unique risk factors for UAS include persistent acidic urine and hyperuricuria, which can also be verified in biochemical parameters (Table 2) and multivariate logistic analysis (Table 3) in this study. Fusobacterium may be involved in the degradation and metabolism of certain short-chain fatty acids (Figure 4), and these metabolic pathways are related to the acid-secreting function of renal tubules. The specific expression of Fusobacterium may induce a pro-inflammatory micro-environment that gout patients do not have.

Whether Fusobacteria promotes the expression of inflammatory factors in renal tubular epithelial cells, whether these inflammatory factors promote the formation of stones caused by urine acidification, and whether the metabolic pathway involved in Fusobacteria can be targeted for pharmacological intervention? With the rapid development of computational intelligence, the application scenarios of artificial intelligence (AI) are becoming richer and richer. In the future, computational intelligence can be applied to pharmacological intervention of gut microbiota to treat metabolic diseases such as UAS. The computer obtained the basic data group of gut microbiota of target patients through meta-analysis of a large number of literatures. Through algorithm improvement, a suitable retrieval model was established to complete the application research of the AI-based gut microbiota intervention model for UAS. The computer intelligently learned the basic data processing in data mining, data processing, data cleaning and so on. Through the analysis, it can be concluded whether pharmacological intervention for gut microbiota of UAS is feasible based on data mining, so that computational intelligence can learn more pharmacological intervention plans on the basis of disease association rules. Research and development of drugs through AI can greatly shorten the time of drug development, improve the efficiency of research, control the cost of research, and improve the efficiency of doctors’ personalized treatment.

Until now, the number of subjects included in this study was not large enough, and the results need to be further verified by expanding the sample size. Second, there was no 24-h urine composition analysis for stone formers in this study. The last but not least, due to financial constraints, collected fecal samples were not tested for short-chain fatty acids and lacked metabonomic analysis.

## Conclusion

Renal uric acid stone formers, especially complicated with gout, often have various types of dyslipidemia, persistent hyperuricemia, and aciduria. The richness and diversity of their gut microbiota were different from the gout patients as well as normal population. *Bacteroides* and *Fusobacterium* were positively correlated with the serum UA level of patients with UAS. The pro-inflammatory bacteria, Fusobacteria, may be related to the pathogenesis of UAS and has the potential to become the biomarker. These findings may provide a novel and non-invasive target for the prevention and treatment of UAS, which requires further large-scale investigation.

## Data Availability

The datasets presented in this study can be found in online repositories. The names of the repository/repositories and accession number(s) can be found below: https://www.ncbi.nlm.nih.gov/Traces/study; SRP332626.
