# Supplementary material for: Association of Gut Microbiota and Biochemical Features in a Chinese Population With Renal Uric Acid Stone
Source: Front Pharmacol. 2022 May 19;13:888883. doi: 10.3389/fphar.2022.888883 (PMC9160931; doi:10.3389/fphar.2022.888883)

**TableS1** Comparison of general characteristics between each group of 16s rRNA

| Variables | Gout  (n=15) | UAS  (n=16) | Gout+UAS  (n=17) | Control  (n=17) | *P* value |
| --- | --- | --- | --- | --- | --- |
| Age,years | 56.93±15.54 | 54.44±15.85 | 55.53±13.06 | 55.06±13.19 | 0.969^#^ |
| Gender(%) |  |  |  |  | 0.998^*^ |
| Male | 12（80.00） | 13（81.25） | 14（82.35） | 14（82.35） |  |
| Female | 3（20.00） | 3（18.75） | 3（17.65） | 3（17.65） |  |
| BMI | 23.88±2.59 | 24.91±3.56 | 25.38±2.67 | 24.60±2.42 | 0.512^#^ |
| Hypertension(%) |  |  |  |  | 0.379^*^ |
| Yes | 6(40.00) | 6(37.50) | 9(52.94) | 4(23.53) |  |
| No | 9(60.00) | 10(62.50) | 8(47.06) | 13(76.47) |  |
| Diabetes(%) |  |  |  |  | 0.911^*^ |
| Yes | 1(6.67) | 1(6.25) | 2(11.76) | 1(5.88) |  |
| No | 14(93.33) | 15(93.75) | 15(88.24) | 16(94.12) |  |

Note: #,Analysis of variance (ANOVA).*,2-sided Chi-square test.

UAS,uric acid stone.BMI, body mass index.

**TableS2** Average nutrient intake from uniform diet for 3 days

| Intake of nutrients | Day 1 | Day 2 | Day 3 |
| --- | --- | --- | --- |
| Total energy(kcal) | 1825.4 | 1784.8 | 1756.5 |
| Protein(g) | 76.2 | 77.3 | 75.8 |
| Fat(g) | 35.5 | 34.6 | 32.9 |
| Carbohydrate(g) | 320.8 | 312.6 | 293.0 |
| Total dietary fiber(g) | 11.2 | 10.8 | 11.3 |
| Vitamin A(μg) | 152.8 | 164.9 | 157.6 |
| Vitamin B1(mg) | 1.18 | 1.09 | 1.06 |
| Vitamin B2(mg) | 1.03 | 0.98 | 1.01 |
| Vitamin C(mg) | 87.5 | 82.8 | 95.7 |
| Vitamin E(mg) | 14.8 | 12.5 | 14.2 |
| Carotene(μg) | 2685.6 | 2496.1 | 2567.2 |
| Ca(mg) | 576.2 | 554.9 | 514.8 |
| K(mg) | 1899.1 | 1826.5 | 1788.3 |
| Na(mg) | 375.2 | 356.8 | 366.1 |
| Mg(mg) | 327.5 | 318.1 | 345.4 |
| Fe(mg) | 25.6 | 22.8 | 27.1 |
| Zn(mg) | 13.0 | 12.5 | 12.7 |
| Cu(mg) | 2.26 | 2.30 | 2.18 |
| P(mg) | 1047.1 | 1120.8 | 1011.9 |
| Se(μg) | 55.5 | 47.2 | 53.9 |

**TableS3** The univariate Logistic analysis of risk factors in each group studied

| Variables | Gout | | UAS | | Gout+UAS | |
| --- | --- | --- | --- | --- | --- | --- |
|  | *P* value | Adjusted OR（95%CI） | *P* value | Adjusted OR（95%CI） | *P* value | Adjusted OR  （95%CI） |
| BMI | 0.612 | 1.03(0.91-1.17) | **0.001** | **1.20(1.08-1.35)** | **<0.001** | **2.38(1.74-3.24)** |
| Hypertension | **<0.001** | **2.63(1.54-4.49)** | **0.004** | **2.37(1.32-4.25)** | **<0.001** | **4.39(1.92-10.02)** |
| Hypertriglyceridemia | 0.154 | 1.(0.81-3.82) | **<0.001** | **4.13(1.95-8.78)** | **<0.001** | **7.84(3.08-19.95)** |
| Low HDL-cholesterolemiamia | **0.008** | **2.38(1.26-4.51)** | **0.016** | **2.34(1.17-4.65)** | **0.004** | **3.76(1.54-9.19)** |
| Hypokalemia | 0.633 | 0.80(0.33-1.97) | 0.797 | 1.13(0.46-2.78) | 0.438 | 1.52(0.53-4.36) |
| Hypocalcemia | 0.908 | 1.03(0.65-1.61) | 0.335 | 1.25(0.80-1.96) | 0.099 | 1.60(0.92-2.81) |
| Hyperuricemia | **<0.001** | **11.62(6.21-21.73)** | **<0.001** | **5.14(2.65-9.97)** | **<0.001** | **201.29(25.73-1.58e-3)** |
| Urinary pH | **0.001** | **0.48(0.32-0.73)** | **<0.001** | **0.39(0.25-0.63)** | **0.003** | **0.33(0.16-0.68)** |

Note: UAS,uric acid stone.BMI, body mass index.

Bold values indicate significant difference.

A

A

**
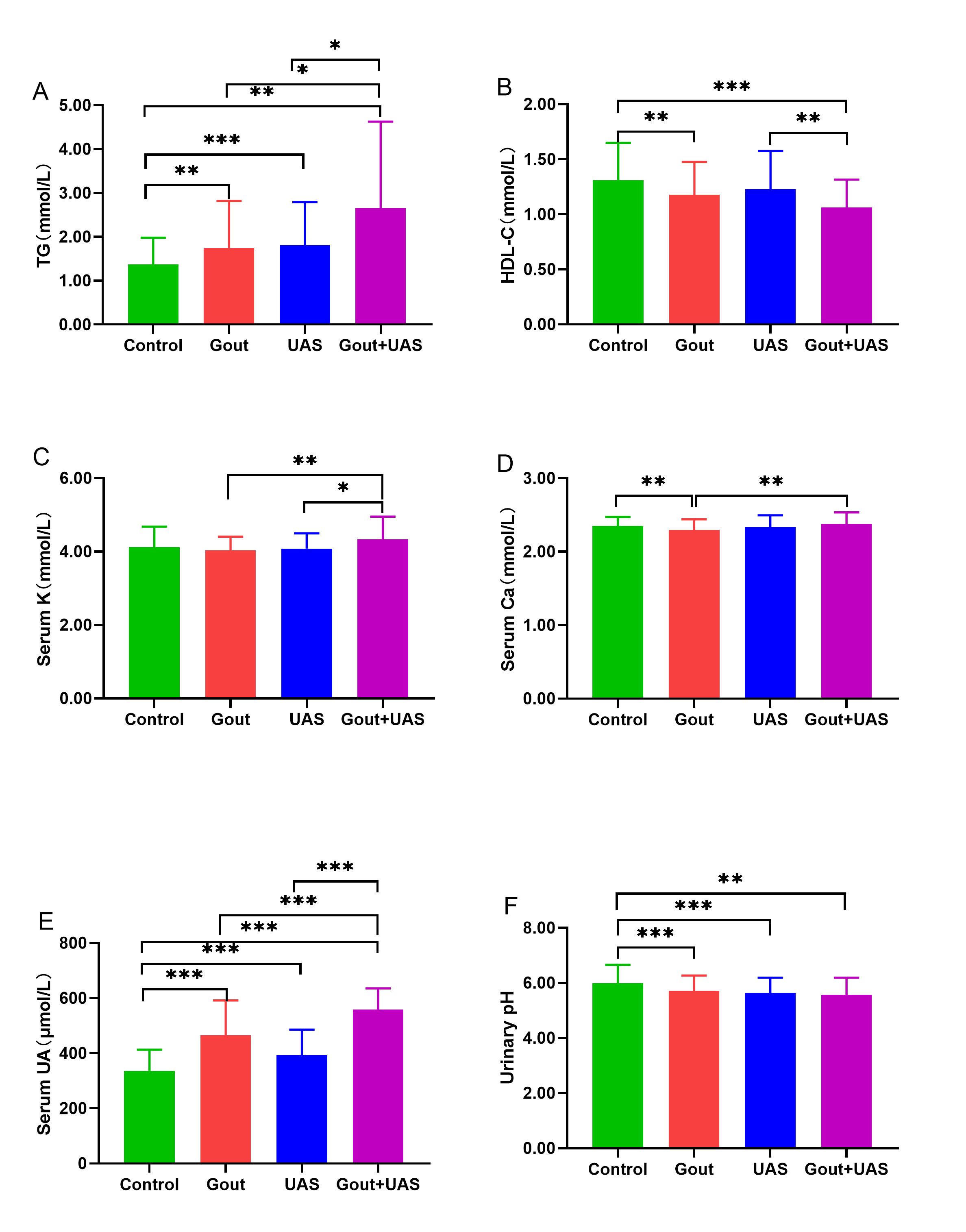
FigureS1** Histograms of biochemical parameters with significant differences between the four groups.Comparisons made using Student’s T test(*,*P*<0.05,**,*P* <0.01,***, *P*<0.001).TG,triglycerides. HDL-C,high-density lipoprotein cholesterol. UA,serum uric acid.


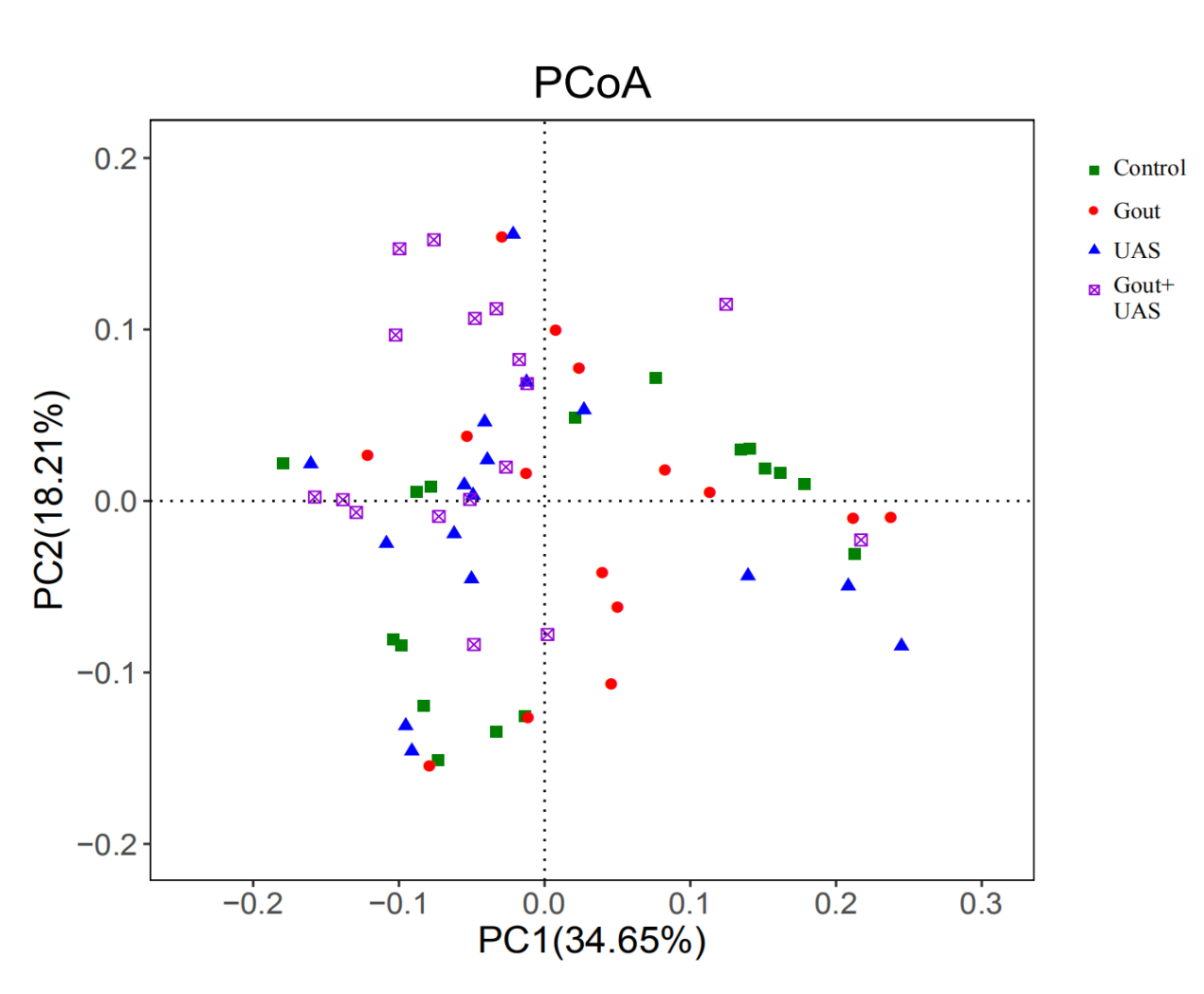


**FigureS2** Comparison of beta diversity of gut microbiota between each group. PCoA score plot based on binary pearson distance at OTUs level revealed classification of Gout patients, UAS patients,Gout+UAS patients and controls.

**FigureS3** Heatmaps of correlation between dominant bacterial abundance and significantly difference biochemical parameters in control group(A),Gout group(B), UAS group(C) and Gout+UAS group(D).The red cell indicates a positive correlation and the blue cell indicates a negative correlation. Stars indicate the degree of significant correlations(*,*P*<0.05,**,*P* < 0.01).TG, triglycerides. HDL-C, high-density lipoprotein cholesterol.UA,serum uric acid. Spearman correlation coefficient values were further adjusted for age and sex.
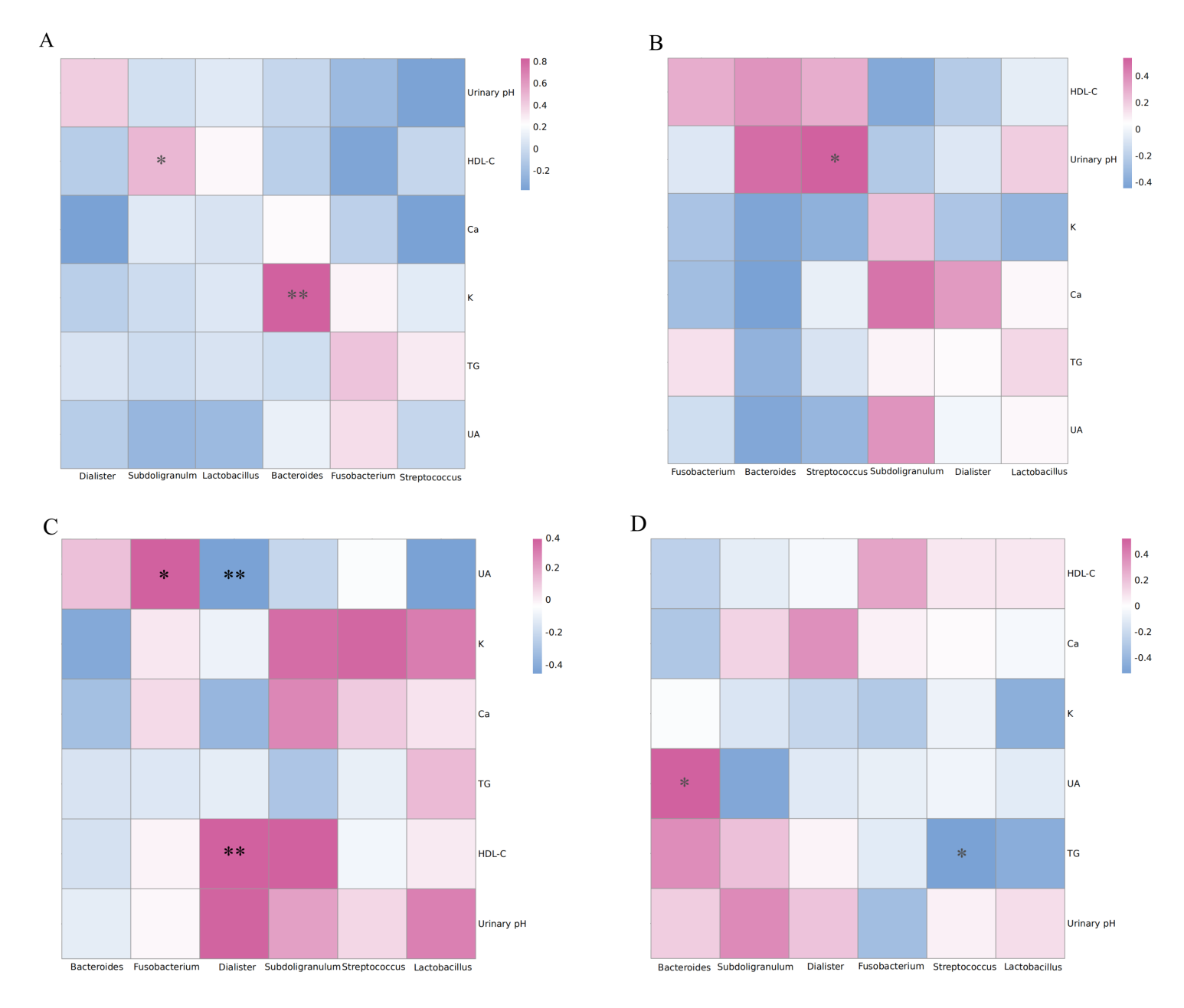

Supplement: Supplementary file 1 [file Table1.DOCX]
